# Wetland Heritage in the Balance: Developing an Exploratory Model for Understanding Local Perceptions of Wetland Heritage

**DOI:** 10.1007/s13157-026-02055-6

**Published:** 2026-03-25

**Authors:** Abbi Flint, Benjamin Jennings

**Affiliations:** 1 University of Oxford, Oxford, England, U.K.; 2 University of Bradford, Bradford, England, U.K.

**Keywords:** Peatland, Cultural Heritage, Cultural Value, Qualitative

## Abstract

Wetlands are well known and researched for the potential biodiversity, ecological and environmental benefits that they contain. In general, these aspects have been referred to as ‘natural’ elements of the wetland landscape. Outside of subject specific studies, such as archaeological projects, less research has explored the cultural aspects of wetland environments, though there is a growing interest in understanding how populations interact with wetland environments. This study focusses on two wetland environments from the UK (Ilkley Moor, Thorne and Hatfield Moors) and uses qualitative research through online questionnaires and interviews using participant selected imagery to prompt discussion and gain insights into public perceptions and understandings of cultural heritage within these wetland environments. We use this qualitative research to propose a model of how wetland heritage is conceptualised and perceived by local users of these environments and to highlight the fluidity between ‘natural’ and ‘cultural’ aspects which are identified as components of the heritage of wetland environments. The research discusses the multiple influences on individual constructions of wetland heritage, with the model offering potential means to explore and work across plural understandings of heritage in landscapes of contested use.

## Introduction

In this paper we offer insights and reflections from qualitative research into local perceptions of peatland heritage from two peatlands in the north of England, conducted as part of *Wetland Futures in Contested Environments (WetFutures). WetFutures* was an international transdisciplinary project which explored approaches to wetland heritage in the Netherlands, UK and Ireland. The project aimed to develop understandings of how ‘wetland heritage’ is constructed through the consideration of inter-related concepts of place, process, communication and people. We use the terms ‘wetland’ and ‘peatland’ to refer to wetlands generally, and the diverse variety of peatland environments included within that (e.g. IUCN UK Peatland Programme 2023; Ramsar 1994). While peatlands are highly important environments for hydrological functions, biodiversity, carbon sequestration, and distinctive forms of heritage preservation, they can often be contested environments; with conservation and preservation efforts sometimes in conflict with extant and historical land-use practices, and with differing understandings of heritage (IUCN UK Peatland Programme 2023; Hazell et al. 2022). This paper aims to shed light on this complexity by exploring the perceptions of local people who have engaged with two UK peatland case study sites: Thorne and Hatfield Moors (THM) in the Humberhead Peatlands and Ilkley Moor (IM) in West Yorkshire. These sites do not represent the full range of wetlands in the UK, however they do reflect two very different contexts in terms of history and topography. IM is an upland peatland, part of the wider moorland landscape known collectively as Rombald’s Moor. In 1925 it was designated as common land and is currently managed by Bradford City Council as a well-used recreational and working landscape. Historically, IM has been used for military training, industrial exploitation (quarrying), recreation and wellbeing (notably since the Victorian period). It is a landscape rich in visible archaeological features dating back to the Late Neolithic/Bronze Age, including stone circles and numerous rock carvings, many with distinctive cup-and-ring marks.^1^ THM are part of a lowland raised mire landscape collectively known as the Humberhead Peatlands, currently designated as a National Nature Reserve (NNR) with public access for recreation. Until the early 21 st century the moors were used for horticultural peat extraction which has significantly shaped the landscape. This connects with a longer history of smaller-scale peat extraction and drainage. Longer timescales of human use of the Humberhead Peatlands are reflected in archaeological remains such as a Neolithic trackway discovered in the peat in 2004 (Chapman and Gearey 2013). The cultural setting and history of IM and THM are thus very different, with different management plans, public accessibility, and use. These differences were the basis of their selection as key study sites for *WetFutures*, and enabled an understanding of perceptions of heritage across wetlands and peatlands of different geo-cultural contexts.

Heritage is a complex and contested concept, difficult to define, and the topic of extensive scholarly debate (see, for example, Smith 2006, 2025; Harrison 2013; Harrison et al. 2020). Heritage may be described through tangible (artefacts, buildings, monuments etc.) and intangible (practices, oral traditions, crafts, folklore etc. also known as ‘living heritage’) dimensions, but it is also about the relationship between these dimensions and the present. As Rodney Harrison (2013, 14) has summarised, ‘heritage is not a ‘thing’ or a historical or political movement, but refers to a set of attitudes to, and relationships with, the past’ which are ‘formed *in the present.’* As such, heritage might be more usefully interpreted as an ongoing process of (co)creation, often entangled with ideas of identity, belonging and sense of place (Smith 2025). Traditional definitions of natural and cultural heritage, and the implication that these reflect a binary separation between nature and culture, have also been the subject of much debate (see for example Lowenthal 2005; van Londen et al. 2019).

…*the idea of natural and cultural heritage as separate domains*,* representing different forms of value and embodying a broader Cartesian dualism through an insistence on the separation of nature and culture*,* body and mind*,* practice and thought*,* tangible and intangible*,* has also emerged as untenable.* (Harrison 2015, p. 27)

Indeed, given the extent of past and present human influence on English landscapes, ‘our historic and natural environments are indivisible’ (Willett 2025, p. 3). Recent frameworks have collapsed this dualism, through more holistic ways of thinking about heritage, e.g. in terms of bio-cultural heritage (Russell 2021) or connectivity (Harrison 2015). Hazell et al. (2022) detailed collaborative working between public bodies with statutory responsibilities for the historic and natural environment in England (Historic England and Natural England), which fed into the ‘England Peat Action Plan’ and recognised the importance of considering the natural and cultural dimensions of peatlands holistically. However, within the UK context, heritage funding streams and financial classifications can embed the division between ‘natural’ and ‘cultural’. The former Ecosystem Services Framework for providing financial quantification of environmental and cultural services was replaced (in 2021 & 2022) with a Natural Capital Approach^2^ and a Valuing Culture Heritage Capital framework^3^. This distinction provides greater potential for cultural heritage services to be given equivalent economic recognition to natural services but could also perpetuate a division between what is cultural and what is natural which can frequently be a highly diffuse boundary (see e.g. Paulissen and van Beek 2024).

Whilst cognisant of these debates, we needed a language to explore the different facets of heritage that are emphasised in our participants’ perceptions of peatlands. Within the research detailed in this paper people described elements which aligned with *perceived-to-be* natural aspects of environments (animals and plants, geology and hydrological systems) and those which imply greater cultural influence (archaeological sites and artefacts, historical land use, folklore and cultural practices). In practice, natural and cultural, and tangible and intangible, may be conceptualised less as discrete categories but instead as shifting and fluid emphases. As Jeff Malpas has noted, as heritage is ‘always configured in relation to the “material” […] there can be no clear or sharp distinction between the natural and the non-natural, the tangible and the intangible’ (Malpas 2008, pp. 203-4) A particular peatland feature – such as a lake or copse of trees – can be perceived by people as natural but have actually originated through cultural actions (extracting peat for fuel or planting trees to shelter livestock). Peatlands continue to be living, dynamic systems of entanglements between human and other-than-human, therefore unpicking the specific influence of single elements is difficult (as Stratigos 2022; has also identified for lake environments). Whilst we acknowledge this complexity, we do sometimes use the terms ‘natural’ and ‘cultural’ as a necessary short-hand for where *emphasis* in perceptions might lie.

Just as there have been, and continue to be, diverse interests and uses of wetlands there will also be different views about wetland heritage; both in terms of how it is conceptualised and how people engage with it. Professional perspectives on wetland heritage may be expressed in scholarly and policy publications (e.g. Gearey et al. 2010), and in formal criteria and designations, such as the National Heritage list for England for cultural aspects and Natural England’s designations for environments such as NNRs and Sites of Special Scientific Interest (SSSI).^4^ As Laura-Jane Smith (2006) has described, alongside these ‘authorised heritage discourses’ are the ways that members of the public think about and engage with ideas of heritage. In this way, there can be plural heritage discourses about peatlands. The aim of our study is to gather the perspectives of local people who already engaged with and had a connection with our two case study sites, to explore how they conceptualised the heritage of these sites and the role, if any, that heritage played in their engagements with wetlands.

Published studies of public views of wetlands have tended to focus on contemporary natural or ecological aspects of these environments, particularly in relation to conservation and ecosystem services (e.g. Scholte et al. 2016; Aggestam 2014; McInnes 2013), which perhaps reflect the emphases on conservation and preservation in later 20th and early 21 st centuries.^5^ The first decades of the 21 st century saw increasing focus on the ecosystem service values that peatlands contribute, particularly in the form of carbon sequestration and water retention within climate change mitigation strategies and policies, and wider ecological concerns around biodiversity (e.g. UK Government 2021). Fewer studies have attempted to understand how people engage with and perceive the cultural dimensions of wetlands, even though they are known to contain a wealth of archaeological, palaeo-ecological and –environmental indicators, and rich intangible heritage. As Flood et al. (2021, 1) have noted, cultural ecosystem services (one of which is heritage) are ‘often under- represented in peatland conservation, management and decision making.’ Tangible forms of wetland heritage may be visible, for instance standing stones and buildings, or ‘invisible’ (contained within the peat), such as bog bodies (Giles 2020). Remains can be macro scale (e.g. artefacts, human remains, structures) or micro scale (e.g. pollen remains) (Gearey and Chapman 2022). These heritage features can be distinctive to a particular peatland’s location and relate to prehistoric activities (e.g. field boundaries, settlement, burials) or historic and near-contemporary activities (e.g. aircraft crash sites, quarrying, agricultural practices). Intangible heritage may relate to folklore, traditional land-use practices (e.g. peat cutting for fuel) or management for leisure activities (e.g. walking). These cultural aspects are as at-risk as the natural if peatlands are degraded (Gearey and Chapman 2022; Milner et al. 2011). When cultural aspects are mentioned in studies of public perceptions, these mainly relate to nature-tourism, recreational and agricultural uses (e.g. Lee 2011; Pueyo-Ros et al. 2019).^6^ However, as Davenport et al. (2010, 719) noted, wetlands are not only seen as important ecologically, but have “historical and contemporary cultural significance to many local people”, which is also highlighted by Hamman (2025) in relation to the Ramsar convention and Baylan (2023) and Paulissen et al. (2022) in relation to creation of meaning.

Informed by the recurrent findings of human remains, the famous ‘bog bodies’ (van Beek et al. 2023), and deposited collections of metalwork ‘hoards’, peatlands have often been interpreted as liminal spaces; between life and death, domesticated and wild (Giles 2020; Bradley 2017). While these interpretations have undoubtedly been influenced by wider cultural perceptions of peatlands at the time of their development, they also indicate that peatland environments have held plural meanings for a long time and have frequently been a place of conflicting interests and uses. Byg et al. (2017) found considerable ambiguity in contemporary public perceptions of Scottish peatlands, leading the authors to suggest peatlands could be viewed as simultaneously good, bad, and ugly. Indeed, reducing this to the most fundamental point, there is no single story of peatlands. Instead, the stories of peatlands are co-constructed and (re)written by those who engage with them. Furthermore, these stories are dynamic: they will develop and change depending on the individual experiences and aspirations of those who engage with them, whether they are local to a peatland and frequently visit, or whether they are more distant and rarely able to access the environment. Consequently, peatlands reflect a palimpsest environment with explicit and implicit heritage assets defined by more than simply their visible presence. A holistic understanding of these heritage assets requires a situated approach which includes how they are perceived by local people who engage with them.

## Methodology

Our research approach was qualitative, employing online questionnaires and semi-structured interviews, and broadly phenomenological (Saldaña 2011) – interested in exploring the meanings and experiences of ‘heritage’ among people who engaged with these peatlands. The research was conducted in 2020-21, during periods of restrictions due to the Covid-19 pandemic, which posed ethical and practical challenges for face-to-face methods, therefore all research was conducted remotely. This part of our research focused specifically on the perceptions of local people who had existing connections with the peatland landscapes, to gain insight into how people familiar with peatlands understood their heritage, and whether that played a role in how they used and valued these landscapes. Wider public perceptions of these landscapes were gathered through literature review (see Flint and Jennings  2020) and analysis of visitors’ online reviews (see Flint and Jennings 2021) and are not the focus of this paper.

The online questionnaires included a mixture of open and closed questions to enquire about: how people currently engage with the peatland; awareness and perceptions of peatlands and their heritage; the challenges they feel face peatlands; and, if/why the peatland is important to them. Questionnaire design was inspired by an existing tool used within the wider *WetFutures* project but was tailored to our research questions and the context of each peatland (IM and THM) and tested and refined through an online cognitive interview with a potential research stakeholder (Beatty and Willis 2007). Target respondents were local people who engaged with the peatlands in some way. Local interest groups for each site were identified through web-searches and key contacts were approached via e-mail to ask them to share the link to the relevant questionnaire with their members. The groups which agreed to circulate the questionnaire had interests in conservation and nature, history and archaeology, exercise, and education. In addition, for IM a local news site and radio station were approached with a press release which they shared, and at THM information boards and QR codes linking to the questionnaire were displayed onsite. All research participants gave voluntary informed consent. The 94 valid responses received (74 from IM and 20 from THM) were analysed using descriptive statistics, and thematic analysis (Braun and Clarke 2006) of open text comments. Respondents tended to be from older age groups, with 81% of IM and 40% of THM respondents aged 60 or older. See Tables 1 and 2 for the distance respondents lived from each site and how often they visited. The differences between sites relate in part to their locations; IM is close to and easily accessible from the town of Ilkley whereas THM are slightly further from urban conurbations.

**Table 1:** Distance that Questionnaire Respondents Reported Living from Each of the Case Study Sites

	Within 1 mile	1–5 miles	6–10 miles	11–15 miles	16–20 miles	More than 20 miles
IM	62.5%	10.8%	14.9%	2.7%	1.4%	8.1%
THM	10%	30%	35%	10%	0	15%

**Table 2:** How Often Questionnaire Respondents Visited Each of the Case Study Sites

	Several times a week	Once a week	A few times a month	Once a month	A few times a year	Once a year	Less than once a year
IM	32.4%	17.6%	14.9%	5.4%	18.9%	1.4%	8.1%
THM	15%	5%	0	15%	35%	15%	15%

Interview participants were recruited via invitation within the IM questionnaire and were selected to reflect a range of ages and genders: three identified as male, one as non-binary, and four as female. The choice of a single site for the interviews was not intended to be representative of all possible perspectives, but to illustrate a range of views and explore in-depth some of the themes which emerged from the questionnaires. Eight people participated in semi-structured interviews: seven online and one via telephone. The interviews explored individual experiences and perceptions of IM and specifically its cultural heritage. Participants were invited to share up to three images they had taken or selected to reflect what IM meant to them. The use of images provided a participant-led place-based anchor within the interviews, in the absence of being able to be ‘in the field’. We had initially intended to use walking interviews (Evans and Jones, 2011) and after moving to a fully online approach we felt that photo-elicitation offered an opportunity to bring aspects of the physical landscape into the interview, which we hoped would prompt rich and situated accounts of participants’ perceptions and experiences (Silver 2013). Interviews were recorded, transcribed, then coded and analysed through an iterative and reflexive process, aligning with Saldaña’s (2011, 223) description of ‘eclectic coding’ focusing on concepts, process, emotion, values and tensions. These were distilled into key themes relating to perceptions of wetlands and their heritage. In the findings that follow, the site-specific questionnaires are referred to as IMQ and THMQ, and IM interview participants referred to as IP.

## Findings

### Perceptions of Wetlands and Their Heritage

Terms such as ‘wetland’ and ‘heritage’ were not always immediately understood by participants: ‘I genuinely hadn’t thought that peat bogs were ‘wetlands’!’; ‘Not sure what ‘heritage’ means. Is it ‘historic use’ or a characteristic historic environment with a particular biodiversity that should be preserved?’ (IMQ). During interviews, some participants sought validation from the researcher that what they were describing was heritage: *‘*I suppose you’ve got stuff like grouse shooting – would that be cultural heritage?*’* (IP6). As well as implying that there were formal definitions of wetlands and heritage, these quotes also speak to the ambiguous and porous nature of these terms in everyday use.

Participants perceived wetlands as both natural and cultural landscapes. Natural elements were described at the landscape-scale in terms of distinctive topography and terrain; most commonly those closely related to study sites – moorlands, ‘peatland, marsh and boggy land’ (THMQ) – but also displaying knowledge of a wide range of UK and international wetlands:

*Norfolk + in Brittany e.g. salt flats*,* Cambridge fens*,* Maas delta*,* moors Derbyshire Yorkshire Scotland and west Eire*,* Somerset levels*,* swamps e.g. in the Caribbean & suppose e.g. Bay of Bengal*,* tundra of Northern Europe/Russia.* (IMQ).

**Fig. 1:**
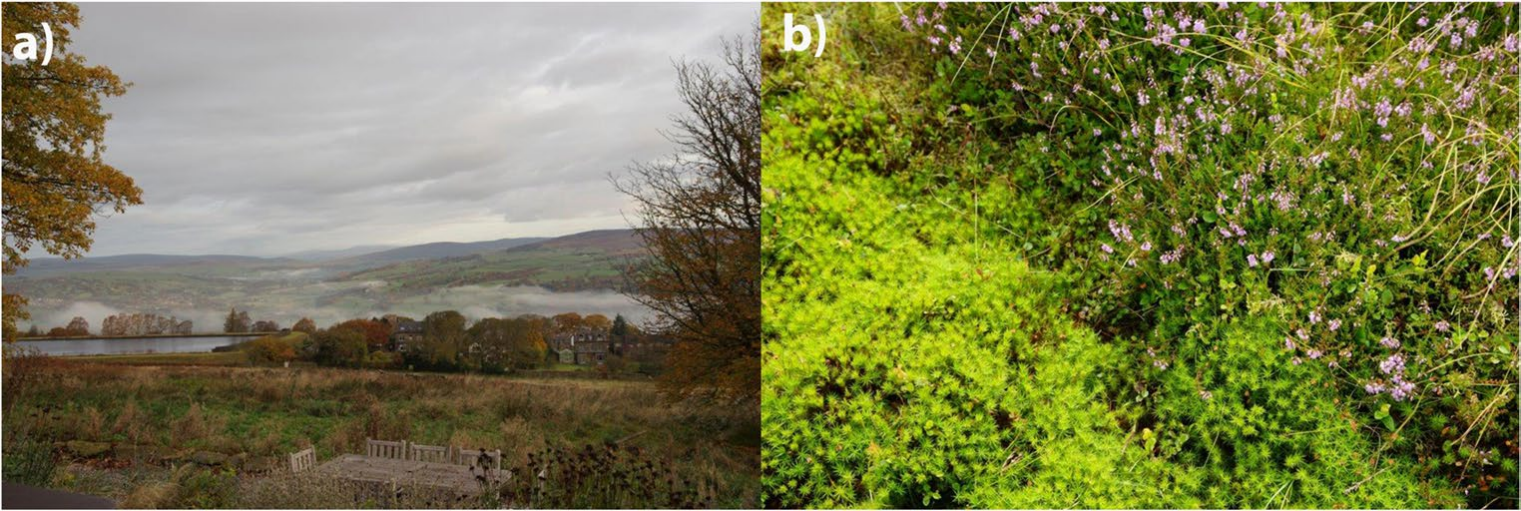
Two Participant Provided Images, One of a Landscape View, Another of Sphagnum Moss, Demonstrate Awareness of the ‘Natural’ Aspects of Wetlands, at Both (Left) Large (Landscape) and (Right) Small Scale (Vegetation) (Photographs Used With Consent of Interview Participant)

Ecological concepts were also used to explain distinctive dimensions of wetlands: as ‘valuable ecosystems […] preserving global biodiversity’, ‘habitat’ (IMQ), and more subjectively as ‘a wonderful wilderness’ (THMQ). Some interview participants also framed IM as ‘wild’ or a ‘wilderness’. Similarly, some photographs provided by interview participants presented an almost idyllic rural view of IM as an unpopulated landscape (Fig. 1). Balanced with this was a sense of fragility; peatlands were perceived as vulnerable to inappropriate use, damaged by historic drainage and peat extraction, and with a perceived lack of wider public awareness of their importance and sensitivity.

Wetlands were also associated with distinctive fauna and flora, including birds (e.g. bittern, nightjar, meadow pipit, raptors), insects (e.g. dragonfly, butterflies, mosquito), amphibians (e.g. frogs and toads), and a range of woodland, moorland and wetland specific plants, e.g. fungi, sphagnum moss, heather, sundew, bilberry and ‘Reed, Rush, Sedge, Willow*’* (THMQ).

*sphagnum moss is quite a well-known wetland … plant species that’s very important for the health of the wetland* […] *It mitigates the rainfall run-off so it can act as flood control and carbon store* (IP2).

Some participants also appreciated wetlands as places where natural and cultural elements are hard to disentangle:

*So*,* what some people*,* and probably me before I was informed otherwise*,* now believe to be a natural environment is actually far from it. It’s almost entirely a man-made landscape […] what we see today*,* which is a series of managed landscapes*,* some of which have been abandoned and nature is modifying them. But […] you don’t have to look very far to see the influence of man on the moor if you know what the signs are* (IP2).

*You get that fabulous kind of wildness*,* you know*,* birds and rocks and heather and*,* you know*,* standing stones and what-not* (IP3).

**Fig. 2:**
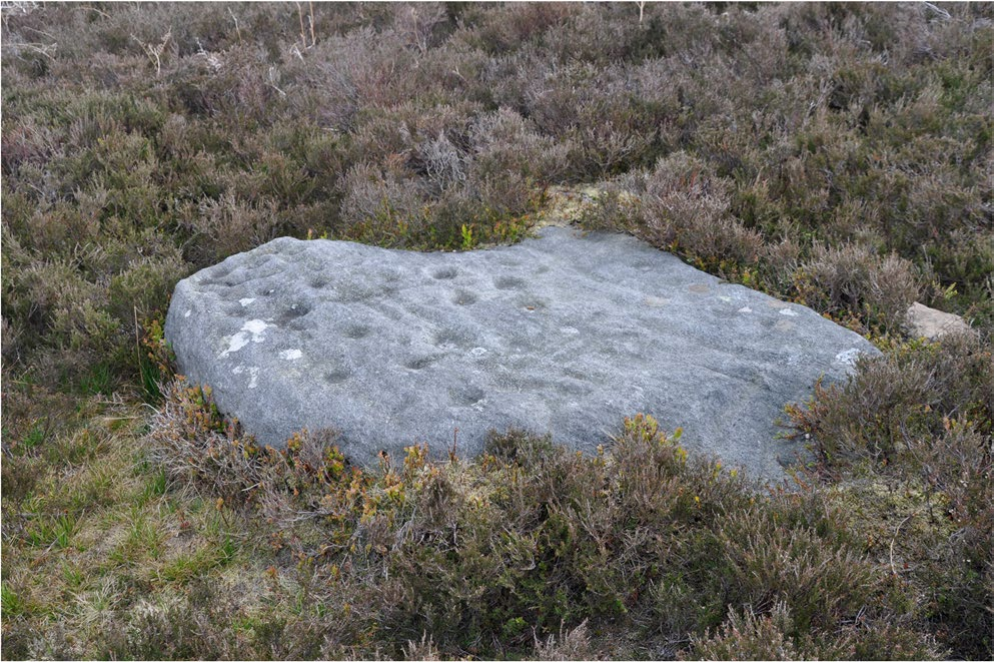
Prehistoric Rock Art visible on Ilkley Moor Provides an Apt Demonstration of the Blurring of Natural Elements with Cultural Influence, Which is Replicated Through Other Aspects Such as Historic Graffiti and 20th Century Art Installations (Photograph by Authors)

As the quote above suggests, some cultural features (such as prehistoric standing-stones and carved rocks) were almost seen as part of the natural landscape and part of the aesthetic appeal of a (perceived) natural landscape (Fig. 2). Interestingly, understanding of the culturally-shaped aspects of wetlands could sometimes lead to dissonance when a feature previously perceived to be natural was there because of human intervention.

*They planted these trees to cover the quarries. And they’re beautiful now […] you wouldn’t really see the quarries now because they’re all sort of grown over […] And I find that quite interesting*,* because I would have thought I only wanted things that were natural*,* but they do look lovely those pine trees up there* (IP6).

Participants displayed a broad understanding of what are considered cultural dimensions of heritage, particularly those which left visible traces of past human presence in the landscape. These included general wetland archaeological features such as crannogs, timber lake-dwellings, Bog-people, Neolithic trackways, prehistoric tools and implements, (THMQ), and, occasionally, ‘Waterlogged physical and environmental remains. Evidence of changing land condition and land use over time’ (IMQ).

In relation to IM, people mentioned visible archaeology such as carved rocks and stone circles, settlement and burial sites, that reflect a long human presence at the site; sometimes referring to specific sites such as the Badger Stone or the 12 Apostles Stone Circle. At THM the features described, which reflected prehistoric human activity, were wooden trackways, whose timber had been preserved in the peat and had been the subject of a recent reconstruction project^7^. More recent heritage structures included buildings (e.g. White Wells bath-house and remains of a school at IM), twentieth-century aircraft crash-sites, and evidence of past activities which had shaped the landscape, including:

- Peat digging (on industrial scale at THM) mining and quarrying, farming, and fishing and hunting (especially grouse shooting on IM);
- Water management and drainage practices;
- Movement and transport – e.g. former industrial transport routes from quarries (IM) and the Crowle Peatland Railway (THM);
- Recreation and tourism – e.g. an early 20th century golf course, Victorian pleasure gardens, footpath networks, and historic graffito (IM).
- Wellbeing activities – e.g. the history of hydropathic houses on the fringes of IM.

These were also perceived as influencing ‘natural’ aspects of the landscape, such as management for grouse-shooting increasing heather coverage and ‘favouring game birds over what are perceived as their predators’ (IP2).

A key part of many participants’ construction of IM’s cultural heritage was its history, stretching across Roman, Viking, Normal, Medieval and Victorian and Modern periods, including the recreational use of the moor and its history as a spa town.

*The history and how the moor’s been used*,* so when I talked about the Victorians used to come out from Leeds on their holiday trips […] that would be the cultural history of it to me. When I talk about the Neolithic stones that Ilkley Moor is famous for and cup and ring marks*,* that would be part of the cultural history of the Neolithic people […] So it’s how the moor has been a background to the lives of other people* (IP5).

People’s understanding of heritage was also shaped by their individual knowledge and experiences. Some had undertaken personal and informal research, others had been involved in historical and archaeological projects such as the designation and mapping of rock art and scheduled monuments (IM), or the reconstruction of a Neolithic Trackway and analysis of palaeo-environmental material (pollen) from THM. These were some of the few respondents who included the less visible environmental archive as part of wetland heritage. Heritage was also expressed through what we might think of as intangible heritage. Some of these were folkloric, such as associations with the Giant Rombald in connection with geological and archaeological features, and stories of ghosts, UFO sightings, and druidic practices at IM. These held a sense of ‘Liminality – dark otherworldly environments where you might meet ancestors, spirits and dark world forces’ (IMQ). Examples of intangible heritage were not always immediately offered in interviews, and when prompted some participants indicated these elements of heritage were not particularly valued or taken seriously; one interview participant described them as ‘daft’, another said:

*Oh yes. The druids haunt Ilkley Moor [laughter] and strange lights have been seen above Ilkley Moor from UFOs and… I try and catch sight of these druids but they’ve never shown themselves to me* (IP5).

Other living heritage included specific place names (toponymy) linked to historical uses and character of sites, e.g. the use of wetland specific language in place names such as ‘carr, Ditch, Drain, Holme, Island, Marsh, Moor, Sewer, Spring, Swamp, Turbary’ (THMQ). A distinctive association for IM was the traditional song, *On Ilkley Moor Baht’at* which, for some who had not grown up locally, was the way they first heard about the moor and for others it was seen as an ‘unofficial Yorkshire anthem’ and a way to connect with regional identity: ‘I feel like, by singing or playing it, I can connect to a lot of other people who are from this area’ (IP1). There was a personal element here too. One interviewee described picking bilberries as a child with their mother, then continuing this with their own children as kind of ‘folk tradition’ (IP4). Another described a new calendar custom of taking a dip in White Wells bath-house (IM) on Yorkshire Day and New Year’s Day. Other interview participants described spiritual dimensions, such as IM being used by druids and for Christian church services.

Almost all interview (IM) participants spoke about heritage in a way that suggested this was an ongoing process. In the main, this was a recognition that it had been a meaningful place for humans from prehistory to the present day. Some explicitly referred to recent additions that were also part of heritage, such as a renovation of White Wells bath house in the last 30 years as ‘a living, you know, historical part of the moor’ (IP7) or the re-use of flagstones from disused mills in footpaths. However, not all evidence of past human activity was valued as heritage:

*I’ll qualify preserving because obviously some of the things that have been done*,* like draining all the moors*,* it’s now understood isn’t a good thing to have done. So I wouldn’t want to retain that just cause it’s there* (IP2).

Some expressed uncertainty about the authenticity of some heritage features, such as the 12 Apostles stone circle (IM), even though this is a scheduled ancient monument (NHL1011763):

*I like the 12 Apostles stone circle […] I think it’s a – it’s not a real*,* genuine stuff – I think it’s a bit of a folly*,* I think it might be a Victorian Folly* (IP6).

Similarly, some participants reflected that what counted as heritage could change over time: for instance, that some carved rocks (Panorama Stones) would have been destroyed in historic building work had it not been for a forward-thinking individual who preserved them. Others questioned whether grouse-shooting was part of heritage, or whether contemporary additions to rock art represent a continuation of activities or modern vandalism:

*[…] on that rock – the Hangingstones one […] there is actually*,* further round*,* a modern one which is beautiful*,* it looks like a lattice*,* kind of*,* a stained-glass window carving or something to me. But it was done in the 90 s and when I went up there with my friend*,* we couldn’t decide whether that was a good thing or bad thing. Cause we were like*,* we can see where they are coming from that it’s changed again*,* but on the other hand*,* you know*,* did they have to do it on this particular stone with a 4000 year old carving?* (IP4).

### ‘The Background To My Life’: Local Engagement With Wetland Heritage

Research participants recognised that these landscapes had plural uses – being ‘a lot of things for a lot of people’ (IP2) - and many engaged with the sites in multiple ways themselves, including walking (sometimes with dogs), outdoor pursuits and sports, general leisure activities (such as enjoying views and having picnics), nature watching, civic activities (such as nature conservation, heritage volunteering, and tackling moor fires), informal learning about nature and history/archaeology (e.g. through guided walks), taking part in religious services, and as an inspiration for creative activities (such as writing and photography).

Participants described their engagements with peatlands, and the value they drew from these, as connecting with heritage in different ways. Peatlands were appreciated at the landscape-scale for their open-views and the aesthetic value, which evoked a sense of escape and tranquility. Participants valued multi-sensory engagements with peatland plants and animals including: hearing frogs at a tarn; the sound of moorland and wetland birds; the feel and smell of bracken when walking; and, eating bilberries; alongside the distinctive smell of peatbogs and moorland air, and the sound of streams. Often these evoked a positive emotional connection to the landscape and contributed to a sense of mental and physical wellbeing.

Participants’ engagements with these landscapes could also be framed as a continuation of heritage as a dynamic process. For some, this was a feeling of connection across time when they were in the landscape, or as continuation of historical recreational uses like walking and picking bilberries with children. For others, it was a direct, intentional action which connected their own interests and identities with heritage, through nature conservation, archaeological reconstruction and volunteering as part of citizen science projects, and personal study. Cultural heritage could be the focus of this intellectual engagement for some: one interviewee described how they had ‘a big interest in local history’ which started in school and continued in their adult life through interests in stone circles and carved-rocks (IP4). Another described themselves as ‘a local historian’ (IP5). These people played an active role in fostering the interest and engagement of others with peatland heritage through writing history and walking guides, and involvement in local projects. They took pleasure in sharing their heritage knowledge with friends, family and visitors, which ‘helps create community’ (IP5) and foster care for the landscape: ‘sharing a bit of our culture with them’ (IP4).

*It has so much history – many of my friends have asked if I can take them and the kids to see some of the carvings* (IMQ).

*I see myself as playing a part*,* a small part in just helping people understand where they live better and thus appreciate it and look after it and thus create meaning* (IP5).

. However, for others, this was only something they reflected on as a result of the interview itself:

*Researcher: […] do you see the way that you use the moor as part of its cultural heritage?*

*IP1: Yeah*,* I’ve never thought of that at all*,* but like I guess everyone is part of history in some sense aren’t we? […] and what I hadn’t even thought about as an aspect of its cultural heritage […] is the heritage of the moor as a place of leisure and hiking. I don’t know how far that goes back.*

Personal connections with peatlands were sometimes reflected through possessive language, and the framing of landscape as ‘home’ or ‘part of my being’ (IMQ)

*I think of myself as an Ilkley Moor lad if you like. From childhood to ancient retirement*,* I’ve been involved with Ilkley Moor and feel it belongs to me […] I belong to it and it belongs to me* (IP8).

*My family are from this area. They farmed it. If we don’t protect it*,* we’ll lose it* (THMQ).

For some, this was a life-long association connecting with key life events and memories: ‘this is the background to my life really’ (IP5). One interviewee was born in a maternity hospital overlooking IM, others described how as children ‘the moor literally was our playground really’ (IP4), and older participants described becoming more actively involved in retirement through volunteering. Positive emotional attachments were expressed through adjectives (e.g. amazing, stunning, inspiring, awesome) describing both natural and cultural aspects. These are landscapes people love and enjoy spending time in, that could be a place of comfort in times of grief, or exhilaration through a sense of exposure and freedom. Encountering peatland heritage prompted feelings of wonder and intellectual curiosity as to how they came about. For instance, IM’s carved rocks were described as ‘fascinating’ and ‘making ‘your spine tingle’, and an Easter church service on IM was described as ‘a profoundly spiritual experience whatever your belief’ (IP3). These comments also reflect connections between people, place and time, that participants described as enabled through engagement with peatlands and their heritage, connecting empathically across time. Engagement with heritage evoked connections at plural scales: with the daily and seasonal, generational lifespans, and the deeper time of history, archeology and geology:

*It’s where generations of my family have played*,* socialised*,* exercised and reflected* (IMQ).

*The history here is just amazing going right back into the Palaeolithic*,* through the Mesolithic*,* through the Neolithic*,* you know*,* you can trace the evolution of humankind here and how it’s formed the landscape.* (IP5).

*there’s a feeling when you’re walking around that you’re treading in the footsteps of people from thousands*,* you know*,* hundreds and thousands of years ago* (IP6).

Encountering heritage didn’t only facilitate connections with the past: an understanding of palaeo-ecology and archaeology could ‘inform the future conservation of important wetland sites’ THMQ). Similarly, ‘peat bog maintenance and reinstatement’ and attempts to raise water tables and clear non-native/invasive plant species were perceived as future-looking. This theme of time, including past, present and future, also links to a dynamic understanding of heritage as something which has developed in the past, in the present and may change again in the future:

*I think…we’re part of that…ongoing*,* you know*,* this is clearly a really important place and we are part of the*,* we’re part of keeping it important and keeping its relevance* (IP3).

The long comment below demonstrates these rich and entangled meanings and engagements with wetland heritage. This respondent draws on cultural and natural dimensions of heritage, their recreational use of the landscape and how that links to a sense of spirituality through providing a connection or ‘association with previous generations that have used/experienced the environment’, and their active involvement in heritage-related volunteering which sparked their intellectual interest and gave a sense of time-depth:

*Hatfield Moors. Though damaged by peat milling it retains a rich wildlife environment home to migrating birds (including the Nightjar). It provides me with the freedom to roam (get my 10000 steps done several days a week) and a chance to come across the Nightjar as well as other wildlife (Hen Harriers*,* Barn Owls*,* Roe Deer etc.). Also in 2004 a Neolithic track was uncovered there which links me spiritually to the past (I took part in the building of a replica Neolithic track on the moors as part of an experimental archaeology project). I also took part in another project […] as a citizen scientist identifying and counting pollen in core samples taken on the Moors. I was fascinated to see through the microscope the pollen of trees growing there thousands of years ago.* (THMQ).

### A Model for Exploring Local Perceptions of Peatland Heritage

**Fig. 3:**
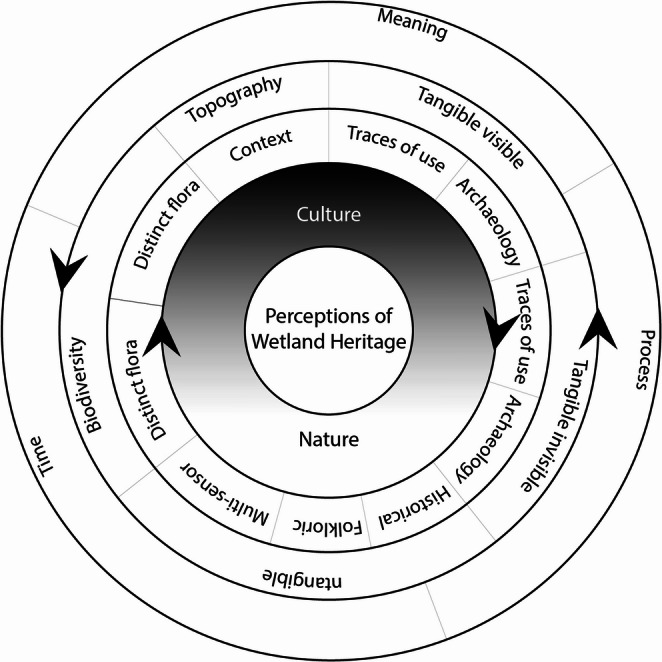
Schematic Model for Perceptions of Peatland/Wetland Heritage

Through our interpretation of interview and questionnaire data, we propose a nested model for exploring perceptions of peatland heritage (Fig. 3).

The inner ring recognises that people’s perceptions of wetland heritage drew on aspects which were perceived as both natural and/or cultural and that the borders of these concepts are permeable and mutable. The influences of cultural and natural agents within wetland environments are complex and entangled.

In the next ring, is our interpretative grouping of the dimensions of heritage that emerged from analysis of our interview and questionnaire data. These include the distinctive landscape features and context, fauna and flora that characterise wetlands, traces of past human presence and activity (archaeological, historical and folkloric), and the multi-sensory nature of engagements with these dimensions.

Mapping these dimensions onto broader heritage categories these may be seen as tangible/visible, tangible/invisible, intangible (living heritage), and relating to topography and biodiversity.

In the outer ring, we frame these understandings within three interlinked concepts. These are the perceived time-depth of peatlands, the notion of heritage as a dynamic, ongoing process, and as something from which people co-create meaning through active intellectual, affective and physical engagements with wetlands and their heritage, connecting with ideas of identity and belonging.

## Discussion

Within archaeological and professional discussions and/or presentations of cultural heritage there is a tendency to over-emphasise the centrality of the visible – tangible objects, material things and assets (Watson and Waterton 2010). This is understandable given the need to rely on the imagination of potentially distant, diverse, and unknown participants to draw in the more intangible aspects of heritage (Alves 2018). Models of valuation of peatlands, explicitly, have focused on contemporary lived experiences, and the emotional and well-being aspects which are facilitated, alongside political focuses on ecosystem services (Flood et al. 2021). Definitions also come from a historic environment perspective, emphasising past human interactions with landscapes (Historic England 2024). Key within these definitions and models of understanding is an emphasis on the division and relationship between cultural and natural, which tend to come from specific disciplines and separate aspects of heritage such as archaeology, folklore, history, and nature. What we found within our research is that people do not necessarily compartmentalise heritage in this way. Instead, our participants’ views resonated with the debates discussed in the introduction; describing heritage in ways that blurred the natural and cultural, reflected complex relationships between the tangible and intangible, and included very personal associations. This reflects a broad understanding of cultural heritage, in line with UNESCO definitions.^8^ In constructing our model, we attempted to reflect this breadth and complexity and asked: what does it look like if these diverse components of wetland heritage are integrated within a holistic model?

Many public and community engagement projects have recognised the value of weaving together environmental and cultural histories (and futures) of peatlands when engaging people with these landscapes. For example, The University of Glasgow’s online exhibition *Bright Edge Deep*, drew together cultural and scientific aspects of peatlands to “celebrate the beauty and biocultural significance of peat bogs.”^9^ In Estonia, Päll and Pungas-Kohv (2024) explored the role of place-lore, which held both cultural and ecological meanings, in navigating conflicting views and interests in mire landscapes. Their work created space for the voices of local people in restoration work and landscape interpretation. Understanding how individuals and collective groups identify and perceive heritage is critical to peatland historic environment and conservation management plans, and the presentation of peatlands, to further engage people with the care of those environments and the heritage within them.

Much of the wider discourse around peatlands and their heritage emphasises their distinctiveness. Prior conceptions of wetland and peatland archaeology have focused both on the uniqueness of the archaeological resources they contain and related excavation methods, but also emphasised they are ‘special’ due to quirks of preservation conditions (Menotti 2012). The difficulty of prospection and remote sensing technologies to identify sites in peatland environments, and thus a reliance on fortuitous discovery of archaeological remains during other peatland use activities (Gearey and Chapman 2022) also contributes to this sense of uniqueness of the archaeological resource in these environments. Our research suggests that some of these distinctive heritage features are appreciated by local people who engage with peatlands, including those associated with natural aspects (such as specific forms of wetland landscapes, plants and animals, the multi-sensory experience of these, and their environmental value) and cultural dimensions (material preserved in the peat, place names, and a folkloric sense of liminality). However, other understandings of heritage (including traces of past human presence and practices, and a sense of ‘wilderness’) were more general, and could just as easily relate to other landscapes. In the same manner that wetland conservation bodies highlight how unique wetland environments are, we have similar occurring for chalk downlands, woodlands, sand dunes, etc. While all these environment types have their own flavour of unique aspects, the heritage elements within our model could potentially be relevant to other environments. This raises questions around whether models of heritage valuation and conceptualisation should be designed with only one specific environment type in mind or whether it is possible to develop approaches which work across different contexts.

Many of the ways in which our participants expressed their engagement with heritage, and what this meant to them, related to connections between people, place and time. Heritage has been recognised as integral to how people create and connect with a sense of place (Malpas 2008; Smith 2006), and with how people mediate the relationships between place and time. As Ashworth and Graham (2005, 11) have noted ‘heritage is the medium through which senses of place are created from senses of time.’ This resonates with Richardson et al’s (2025, 40) notion of local heritage connectedness: ‘a bond that encompasses relations with place and time, present and past, and the array of tangible and

 intangible aspects of heritage.’ Some participants actively invested in peatland heritage through volunteering and research. As the examples of these were both formal (e.g. through joining existing groups and activities such as laying flagstone footpaths) and personal and informal (e.g. writing about local history and archaeology for family and friends), not all these kinds of heritage ‘work’ were obviously visible. Studies like ours bring these contributions made by local people to the surface. Our research also provided examples of the complex ways in which engagement and connection with heritage were valued by local people, in relation to diverse aspects of their lives: mental and physical wellbeing; providing senses of space, spirituality, belonging, and perspective; aesthetic beauty; and, prompting curiosity and wonder. For those who took an active role, heritage knowledge could also be a form of social capital.

Our experience is that enquiring into local perceptions of peatlands provided space for bottom-up explorations of the heritage dimensions of these specific wetlands, highlighting dimensions of heritage we had not initially included and aligning with ideas of valuing ‘heritage from below’ (Robertson 2012). Encouraging participants to describe their understanding of different aspects of heritage, prompted them to reflect on their existing understandings and in some cases articulate a desire to learn more. For instance, one interview participant spoke about wanting to understand how to ‘read’ the landscape heritage of IM (Fig. 2). It seemed that asking about people’s knowledge and awareness of heritage can both surface additional heritage aspects professionals may not have considered, and spark people’s curiosity.

Our model was developed through research focused on public perceptions, meaning a professional perspective has been omitted from the model in the current form. While this may be a limitation of the model, it also permits a bottom-up as opposed to top-down approach; rather than us as researchers determining what aspects of heritage should be considered, general users and non-professionals have identified what they perceive to be heritage in peatland environments. Listening to and valuing public perceptions provides opportunities for conceptions of peatland heritage to be enriched and broadened beyond a single authorised heritage discourse to encompass plural, evolving and co-constructed heritages of peatlands (Smith 2006). This approach values both the expertise of scholars and practitioners, and the local perspectives of people who engage with peatlands in their everyday lives. We do not suggest that either perspective is better, but that both are needed in collaboration to better understand wetland heritage and manage this finite resource for current and future generations.

### Where Next?

A key consideration as we have reflected on our findings and developed our model has been the contextual nature of the study – drawing on research into two case study sites from the UK and informed by the wider *WetFutures* project – and we recognise that not all peatlands will have similar emphasis within their heritage. As such, we have taken a relational approach, allowing elements to inter-link and complement one another, promoting a holistic interpretation of peatland heritage. In doing so we aimed to provide space for expressing localised interpretation and exploration of these elements. However, we are also curious to discover whether it is possible to make a model which remains relevant whilst being broad enough to be applied to a variety of sites. As such, we offer some emerging thoughts on possible ways the model could be adapted to explore perceptions of wetland heritage.

Within our questionnaire and interview responses there were often hints at the tensions which can occur in environments, not just of peatlands but of all types, related to the differing aims, uses, and ultimately the underpinning concepts of heritage which those uses reflect. Participants highlighted familiar issues around, for instance, balancing different forms of recreational usage (such as walking, grouse shooting and mountain biking) with the protection of the rich natural and cultural heritage of peatlands. There are interesting complexities at the individual level, for instance, some people described valuing public access to peatlands but also held idealised views of them as places for nature without other people present. Thus, recreational *over-use* was perceived as problematic.

The model does not offer a direct means to begin resolving these issues of conflict and contest but does offer a way of presenting the plural dimensions of heritage in the round, without imposing hierarchy or judgement. Looking at the model as a whole, with these human and other-than-human dimensions side by side, along with exploring contemporary ways of engaging with peatlands as part of their heritage, may offer a starting point to open-up inclusive conversations about different stakeholders’ perceptions of wetland heritage and to facilitate discussions around tensions and how they might be balanced. This can build upon the relational aspects of heritage, as identified in the model and by others (e.g. Fish et al. 2016; Flood et al. 2021) and develop more inclusive approaches to how dimensions or heritage are formally valued. Turning our theoretical model into active solutions for resolving contested use of environments is beyond the scope of this paper, but the model could be used in further research or public consultation to begin reframing issues of conflict and contestation to those of complexity, negotiating difference, and balance. By mapping understandings of heritage against the elements of the model, and comparing emphases both within and between sites, it could serve as a tool to raise awareness of the diverse forms of heritage at peatland sites, explore the complex reasons behind the value and concepts of heritage and facilitate discussions of balance in terms of negotiating difference. Given the small-scale and focused nature of the study which has informed our model, it would benefit from the input of other studies of public perceptions of peatlands. This could be conducted through further qualitative research, or as part of public consultation in other peatlands. We invite researchers to adopt, adapt, and reframe our model as wished, to further explore the conceptualisation of wetland heritage amongst diverse user groups.

## Data Availability

The research data generated during the current study are confidential, as per ethical approach.

## Footnotes

1 A gazeteer produced by Ilkley Archaeology Group and West Yorkshire Archaeology Service (1986) details 297 carved rocks across the wider Rombalds Moor and the Archaeology Data Service website describes a community project (CSI Rombalds Moor, 2010-13) gathering over 500 records, including 42 new discoveries.

2 See information provided by the UK Government Department for Food & Rural Affairs https://www.gov.uk/government/publications/enabling-a-natural-capital-approach-enca-guidance/enabling-a-natural-capital-approach-guidance.

3 See information from the UK Government Department for Digital, Culture, Media and Sport https://www.gov.uk/government/publications/valuing-culture-and-heritage-capital-a-framework-towards-decision-making.

4 The National Heritage List for England can be accessed on Historic England’s website here https://historicengland.org.uk/listing/the-list/ and more information about Natural England can be found here https://www.gov.uk/government/organisations/natural-england.

5 See Flint and Jennings (2021) for a more detailed summary of this literature,

6 A brief overview of this literature is included within Flint and Jennings 2021.

7 More about this project is available here: https://ioahc.net/project/neolithic-trackway/.

8 See https://whc.unesco.org/en/glossary/224/.

9 The project website is here: https://brightedgedeep.arts.gla.ac.uk/.
